# Community engagement in the Faculty of Health Science: A concept analysis

**DOI:** 10.4102/hsag.v25i0.1403

**Published:** 2020-11-26

**Authors:** Vistolina Nuuyoma, Agnes Makhene

**Affiliations:** 1 Department of Nursing, Faculty of Health Science, University of Johannesburg, Johannesburg, South Africa

**Keywords:** concept analysis, community engagement, community service, higher education, health science

## Abstract

**Background:**

Community engagement has been given different interpretations by scholars and organisations; in addition, current scientific literature has not reached a consensus on how it is defined. This difference in conceptualisation may lead to confusion regarding the meaning. The researcher observed that academic staff from the Faculty of Health Science at an institution of higher education in Namibia are not certain of what counts as community engagement. This has led to some activities from the faculty being cancelled from the institutional review reports as they were not recognised as community engagement.

**Aim:**

The aim of this article is to describe the concept analysis of community engagement.

**Setting:**

the study took place at a faculty of health science at a university in Namibia.

**Method:**

Concept analysis was done in accordance with the eight steps of the Walker and Avant model. A literature search was conducted to capture all potential definitions and uses of community engagement. A total of 225 definitions and uses of community engagement were recorded and used in the concept analysis. A list of definitions and uses of the concept of community engagement were documented with their citations, in a table with three columns. The first column (analysis) consisted of the identified definitions and uses of community engagement from the relevant literature. The second column (synthesis) consisted of reduced statements of the content presented in the first column. The third column (derivation) consisted of the final reduction into categories and connotations derived from the second column.

**Findings:**

Three broad categories were revealed as findings: (1) the antecedents of community engagement, which included community challenges, health inequalities, societal needs and the need for a social responsive approach in education, research and services; (2) a three-phase process of community engagement; and (3) the outcomes of community engagement. A theoretical definition and a conceptual map for the concept of community engagement were drawn from the findings.

**Recommendation:**

The results of the concept analysis of community engagement will be used to develop strategies for its facilitation in the Faculty of Health Science.

## Introduction and background

Community engagement is one of the core functions of higher education institutions and is usually included in their mission and vision statements (Gorski & Mehta 2016:108). The concept of community engagement is influenced by the work of Boyer in 1990, on the broader view of engagement scholarship (Sobrero & Jayaratne 2014:126). During the last 10 years, community engagement at many higher education institutions has become more officialised and bureaucratic. This can be seen through the formation of community engagement centers and monitoring systems at many institutions (Rosing 2015:149).

Community engagement is composed of the concepts ‘community’ and ‘engagement’; each can be understood differently across theoretical traditions. In this research, ‘community’ refers to a spatially or socially isolated group with particular shared properties such as residences, cultural practices and beliefs (Ramsbottom et al. 2017:415). In contrast, ‘engagement’ refers to involvement or being committed to a task (Merriam-Webster Dictionary 2017). Engagement is a continuum consisting of five stages: outreach, consultation, involvement, collaboration and shared leadership (CTSA Consortium 2011:3). Different scholars and organisations have given various interpretations to the concept of community engagement (Ramachandra et al. 2014:156). According to Adebayo et al. (2018:475), the current scientific literature has not reached a consensus on the definition of the concept ‘community engagement’. Most specifically, it seems to be poorly defined in the context of health science education. This difference in definition of the concept may lead to confusion regarding the meaning of community engagement (Adebayo et al. 2018:475). Therefore, community engagement was selected for analysis because of its complexity and ambiguousness and the need to clarify it.

It is necessary to clarify the concept of community engagement because it plays a vital role in the training of health science students. This is because students exposed to community engagement develop into competent and caring practitioners who are aware of the health disparities in their community (Thomas & Smith 2017:63). Community engagement is achieved when students interact with and render services to culturally varied patients and those at risk for health discrepancies. Moreover, the Centers for Disease Control and Prevention (CDC) acknowledged community engagement as a foundation of efforts to improve public health (CTSA Consortium 2011:4). However, the educational models used in medical training and amongst other health professionals focus primarily on hospitals, health centres and clinics, thereby seemingly promoting curative interventions. This could be because of lack of clarity of the concept of community engagement. Students trained via these models tend to understand healthcare as episodic and procedural (Smith et al. 2013:1139). They are inclined to be hospital-centred and insufficiently equipped to manage diseases whose effective cure is prevention and upholding lifestyles that repel diseases (Smith et al. 2013:1139). Smith et al. (2013:1139) further indicated that Health Science curricula should be social and preventive, but not individual and curative.

Concept analysis as a method of inquiry is capable of adding value to the existing body of knowledge in nursing. This is because it allows researchers to distinguish related concepts, refine ambiguous concepts and construct operational and theoretical definitions (Walker & Avant 2014:164). The available concept analysis methods include a model of the systematic elaboration of terminologies based on the ideas of Picht and Draskau (1985) as explained by Nuopponen (2010:7), Nasi’s four elements of concept analysis (Nuopponen 2010:8), Walker and Avant’s eight-step concept analysis method (Walker & Avant 2014:166) and Wilson’s (1963) classic 11-step concept analysis procedure (Walker & Avant 2014:165). The Walker–Avant method (2014:166) is a modification of Wilson’s classic 11-step procedure. The modification was suggested because the eight-step method was found to be adequate to capture the essence of the process. Although criticised for being too realistic and too simplistic (Walker & Avant 2014:179), Walker and Avant’s eight-steps concept analysis is one of the regularly used methods in nursing. This is because the steps are arranged logically in a consistent manner, and they are defensible (Schiller 2018:249). This was therefore selected as a method to analyse the concept “community engagement”.

### Problem statement

There are many routes through which community engagement is conducted in the context of higher education, not only via one process. Community engagement can be conducted via service learning, community-based research and participatory action research (Esau 2015:69; Ross & Stoecker 2017:7). In addition, other methods of community engagement include distance education, outreach and professional community services, service internships as well as community-based projects in the form of directed study or extra-credit modules (Bandy 2018). Despite the many methods of community engagement available at higher education institutions, faculty members are not keen to participate in community engagement, as it is not aligned with the standards for promotion and tenure at an institutional level, and there are no rewards given in recognition of participation (Gorski & Mehta 2016:110). The first author works as a lecturer at an institution of higher education and observed that academic staff in the Faculty of Health Science are not certain of what counts as community engagement when completing their appraisal forms at the end of an academic year. The same observation was also made at the business review of the institution, where some activities from the faculty were cancelled from the review reports, as they were not recognised as community engagement, which posed a problem. There appears to be confusion, ambiguity and a lack of clarity about the meaning of the concept ‘community engagement’; therefore, the research question is, what is the meaning of the concept ‘community engagement’?

### Research purpose

The purpose of this research is to clarify the meaning of the concept ‘community engagement’.

### Research objective

The research objectives are to:

1. identify the uses and connotations of the concept ‘community engagement’
2. provide a theoretical definition of ‘community engagement’ and to develop a conceptual map for it.

## Research design and methods

Concept analysis was conducted in accordance with the basic principles of Walker and Avant’s eight-step model (Walker & Avant 2014:163). This was underpinned by constructivism as the researcher’s paradigmatic perspective. In addition, the analysis, deduction, derivation and synthesis were incorporated as the reasoning strategies. The eight steps are elaborated below.

### Select a concept

The concept ‘community engagement’ was selected because it is complex, ambiguous and still needs more clarity.

### Determine the aim or purpose of the analysis

The purpose of the analysis was to clarify the meaning of the concept of community engagement and develop a theoretical definition. This will be used to develop strategies to facilitate community engagement in the Faculty of Health Science.

### Sample and sampling method: Identify all uses of the concept that you can discover

Dictionaries, thesauri, encyclopaedias, conference papers, research reports, journal articles and books were consulted to search for uses of ‘community engagement’. This was done via library databases and Internet searches. The online library databases that were most instrumental are Medical Literature Analysis and Retrieval System Online (Medline), Cumulative Index to Nursing and Allied Health Literature (CINAHL), Educational Resource Information Centre (ERIC), dissertations and theses. Google, Google Scholar and citations were used to complement the search. From the literature reviewed, the concept of community engagement featured as part of the cognitive processes in learning, decision-making and collaborative activities. Moreover, some literature indicates ontological use of the concept of community engagement. There were differences observed in terms of the contextual use of the concept ‘community engagement’; for example, it is expressed as a practice, a networking activity, a process and a continuum of activities in some contexts. The theoretical sample contained 225 (*N* = 225) definitions and uses of community engagement.

#### Inclusion criteria

The following criteria were used to select the literature for concept analysis:

1. literature in the form of dictionaries, thesauri, encyclopaedias, conference papers, research reports, journal articles, theses, dissertations, print and e-books
2. literature published between 2009 and 2019
3. literature containing one or all of the key concepts ‘community’, ‘engagement’ and ‘community engagement’ in the title or body.

### Data collection method: Determining the defining attributes

The researcher tabulated a list of definitions, the uses of the concept of community engagement and its defining attributes, and their citations in three columns (Schiller 2018:250). The first column is titled ‘Analysis’, and it consists of the identified definitions and uses of ‘community engagement’ from relevant literature. The second column is titled ‘Synthesis’, and it contains a further reduction of the key words from the first column. Thirty-five (*n* = 35) statements were recorded in the second column. The third column is titled ‘Derivation’, and it consists of a final reduction into categories and connotations from the second column. Derivation was done by reading the statements in the second column several times, whereafter they were further reduced and rearranged to obtain categories of defining attributes.

### Data analysis

#### Identify a model case

The researcher constructed a model case using a real-life, educational and health-related example to illustrate community engagement and to clarify its meaning. The model case incorporated the defining attributes identified in the preceding step (Step 4) of the concept analysis.

#### Identify borderline, related, contrary, invented and illegitimate cases (additional cases)

The borderline, related and contrary cases are listed below; however, no invented and illegitimate cases were described, as the model case was descriptive enough to understand the defining attributes of community engagement. Not describing the invented and illegitimate cases in this study is not unusual because most concept analysis reports are likely not to report on them, especially in nursing science (Nuopponen 2010:10; Schiller 2018:252).

**Borderline cases:** Examples of cases related to community engagement would be *promise, appointment, employment, booking, community-based healthcare, community-based learning, research data collection in community, community meetings* and *university open days*.

**Related cases:** Related cases of community engagement are *involvement, interaction, empowerment, development, sharing, extension* and *outreach*.

**Contrary cases:** Contrary cases of community engagement are activities involving non-engagement between a service provider, stakeholders and recipients, and withdrawal from arranged activities shows an absence of engagement.

### Identify antecedents and consequences

Antecedents are defined as occasions or instances that must happen or be in place before the existence of the concept under study (Walker & Avant 2014:173). Consequences are defined as occasions or instances that happen as a result of the existence of the concept under study. In other words, it refers to the outcomes of the concept (Walker & Avant 2014:173); therefore in this study, the consequences of community engagement are referred to as the outcomes of community engagement. The antecedents and consequences are described in the ‘Findings and discussion’ section.

### Identify empirical referents

Empirical referents indicate the presence or existence of actual phenomena that demonstrate the occurrences of the concept itself. In the majority of concept analysis findings, the defining attributes identified are identical to the empirical referents (Walker & Avant 2014:174). The empirical referents of community engagement are social responsiveness, service learning, capacity building, partnership and participatory action research.

### Ethical consideration

Ethical clearance and permission were obtained from the Faculty of Health Science – Higher Degrees Committee (HDC-01-31-2017) and Academic Ethics Committee (REC-01- 40-2017) of the University of Johannesburg on 02 June 2017 and from the Research Ethics Committee (SC/358/2017) of the University of Namibia (project research number REC-241112- 035) on 20 November 2017.

## Findings and discussion

The findings of the concept analysis of community engagement revealed three broad categories: the antecedents of community engagement, the process of community engagement and the outcomes of community engagement. The theoretical definition and conceptual map of community engagement are presented and discussed in this section.

### Antecedents of community engagement

Community engagement does not take place in a vacuum; there must be a situation such as community challenges, health inequalities, societal needs or a need for a socially responsive approach in education, research and services. The community challenges include any difficult situation that affects the wider population, the community as a whole or a group of individuals in the community. It is noted that there are several layers of communities, but each has its own challenges, structure and interest (Cherrington et al. 2018:7). Community challenges may be identified by the community members themselves, faculty members and students. The second antecedent is health inequalities, which is defined as variances in the health status or in the spread of health determinants amongst different population groups (WHO 2018:Online). Depending on the situation, the extent of inequality may be noted to give information on unique situations and set priorities within a given population (Hosseinpoor, Bergen & Grove 2018:655). Some health disparities are related to biological differences, and others may be related to the external environment and conditions mainly outside the control of the affected individuals (WHO 2018:Online).

Societal needs were also identified as an antecedent; these refer to the needs of society as a whole. According to De Haan et al. (2014:126), societal needs are divided into three categories, and they all need to be fulfilled to facilitate liveability in society. The first one comprises existence needs, which include the need for shelter, health, security and material for sustenance such as drinks and food (De Haan et al. 2014:127). This incorporates the first and second levels from Maslow’s hierarchy of needs, which are physiological needs and safety and security needs (Berman, Snyder & Frandsen 2016:476). The second societal need is the relatedness need. This entails the need for interaction, social cohesion, ecological health, knowledge and beliefs, beauty and pleasure, and comfort and convenience (De Haan et al. 2014:127). In Maslow’s hierarchy of needs, it incorporates the needs for love and belonging, which are the level 3 needs (Berman et al. 2016:476). The last societal need is the growth need, which encompasses needs relating to identity, social justice, intergenerational equity, culture and identity, purpose and expression, influence and respect, and freedom and autonomy (De Haan et al. 2014:127). Understanding the societal needs entails extensive exploration of different sources to obtain insight into the pressing needs and expectations of community members (Cassi et al. 2017:1098). When students and academic staff engage with the community, societal needs are identified via community assessments, and then services, information and material supports are provided to attend to the needs. The fulfilment of societal needs is achieved in partnership with community members.

Social responsiveness is a concept used as an umbrella term that denotes all types of engagement with external non-academic populations (UCT 2018:Online). This is also an antecedent of community engagement. The concept encircles engaged scholarship activities involving students and academic staff in the form of providing community service, as well as professional engagement, which entails the application of their professional expertise. A university that conducts such activities is said to be a socially responsive institution, which means it is socially beneficial, generally reachable and structurally flexible (David 2017:184). The process of community engagement requires participants to be socially and culturally responsive to reach out to minorities and disadvantaged individuals. Community engagement activities seek to identify and also find solutions to the challenges affecting community members and to develop the leadership skills of health professionals (Vargas et al. 2012:23). Therefore, being socially responsive may be interpreted as an indicator of the presence of community engagement. According to Coetzee (2012:510), for community engagement to be effective, the facilitators need to understand all elements of community life and understand their behaviours, to determine the immediate and long-term needs. In that way, the engagement activities will be socially responsive. Thus they will address not just one element of the community but will influence or touch the lives of many individuals. The need for a socially responsive approach in education, research and services means that the institution of education should recognise the need to design, implement and evaluate their teaching and learning activities, research and service to the community in relation to the social issues within the community. In their daily operations, academic staff must recognise the need to align their core businesses to the needs of the population they are located in and serving.

### Process of community engagement

The process of community engagement, extracted from the findings of concept analysis, consists of three phases as discussed below.

#### Phase 1: Academic staff and student possession of knowledge of societal needs, community issues and developmental challenges requiring attention in the community

Societal needs were discussed under the antecedents of community engagement. For community engagement to take place, the first step is for academic staff to have knowledge about the societal needs. This is to enable the institution to respond to the societal needs (Navickas & Kontautiene 2015:47). The community issues identified in phase 1 of community engagement are inclusive of community problems and any other issues that need the involvement of academic staff. Phase 1 is triggered by the notion of university contributions to the public good, requests and academic staff and students’ possession of knowledge and skills in health sciences.

#### Phase 2: Community engagement as a university societal role, scholarly activity and an experiential approach

Phase 2 of community engagement assisted the researcher in understanding how community engagement is conducted in the Faculty of Health Science. During phase 2, academic staff engage with their communities as part of the university societal role; in addition, they engage in scholarly activities and an experiential approach. Community engagement is a university societal role because academic staff at the institution of higher education are committed to cultivating a wide relationship outside academic outcomes (Bainbridge 2011:4). That means community engagement also involves the students, not only the academic staff. In community engagement, the academic staff shift away from the agendas of teaching and producing scientific research outputs that are not linked to the society. The role that the university plays in community engagement is the move towards focusing on social and economic objectives through co-creating knowledge and skills with the public good in mind (Escrigas et al. 2013:xxxv). Academic staff not only seek to fulfil curriculum requirements but should also be involved in issues that are outside the university and outside their classroom. For instance, in the Faculty of Health Science, academic staff are expected to be involved in health awareness campaigns and also to conduct different disease prevention initiatives in conjunction with community members (Preece 2017:2).

The process of community engagement is a scholarly activity in that it cuts across the missions of teaching, research and university services (eds. Bringle, Hatcher & Joness 2011:29). Academic staff are involved with the external community and stakeholders in collaborative academic teaching and research to address critical developmental issues. This is not only for the benefit of community members, but for academic staff to enrich their teaching, learning activities and the research objectives of the higher education institutions (UNISA 2018:Online). Community engagement as a scholarly activity helps to prepare the academic staff for their teaching roles through advancement of knowledge and skills in their field to guide the students. This is because the community engagement process also involves consultancy, continuing education and collaborative research in which the academic staff may also participate, not only the students (Mugabi 2015:193). In addition, there is an opportunity for enquiry-based learning in community engagement, whereby the academic staff and students may conduct fieldwork for further exploration of aspects of their field of study (Miller 2013:47).

Experiential learning is an approach that is fully established in Health Science curricula, whereby learning takes place by doing or as a result of experience rather than listening to other people or reading about it (Hughes & Quinn 2013:28). The community engagement process may be considered an experiential learning approach because it enables the active involvement of students whilst bringing about a greater degree of interaction in the learning process. The students are afforded some autonomy, flexibility and exposure to the content relevant to the learning experience. Students are allowed to partake in curriculum-related volunteerism services, internships and some field research courses, which all support experiential learning (Strom & Whiteford 2013:86). These activities are appropriate for health science students so they can be exposed to real-life experiences in communities and health facility settings, to gain understanding and construct their own knowledge in the field. For health science students the process of constructing their own knowledge is facilitated by reflection on the experience; therefore academic staff encourage students to continuously write reflective notes with every experiential learning encounter.

#### Phase 3: Values promoting community engagement

Phase 3 of community engagement encompasses values that are identified as enhancers of community engagement. The values identified are commitment, partnership and reciprocity, inclusiveness and informing, relevancy and belongingness, quality, flexibility and sustainability, connectivity and efficient communication, documentation, monitoring and evaluation, active participation, promoting awareness of activities and civics importance.

The process of community engagement is enabled by key aspects, and the *commitment* of participants is one of them (Giloth 2018:32). When there is commitment in the process of community engagement, key stakeholders portray dedication and loyalty in their contributions. Moreover, the commitment in partnership enhances personal contact and boosts a knowledge-sharing culture amongst the people involved (Marlier et al. 2015:4). Partnership is core to nurture trust amongst participating members and ensures their willingness to engage during the activities and to invest their time and effort (Quillinan et al. 2018:119). In community engagement, the concept of reciprocity is used to describe partnership between stakeholders and engaging institutions and is used for classification and categorisation purposes. For example, the Carnegie Foundation acknowledged reciprocal partnerships as a key criterion for the community engagement classification (Schaffer & Hargate 2015:60). Reciprocity is important in the community engagement process because it allows mutual benefits in terms of learning and research (Cherrington et al. 2018:6; Smith, Else & Crookes 2014:845). Successful reciprocal practices in community engagement involve consideration of all planned activities to benefit all parties concerned.

Inclusiveness is one of the core values identified by land-grant universities for facilitating engagement between higher education and society for the development of individuals, families and communities. Inclusivity in higher education engagement facilitates the subject matter taught and the research conducted to take account of topics that are relevant to society (Fitzgerald & Simon 2012:38). That means that university researchers and practitioners work closely with community members to invent innovative and viable solutions to societal problems. Therefore, inclusiveness signifies fit for purpose (Glandon et al. 2017:1458). Belongingness is another value that promotes community engagement. This is because a sense of belongingness promotes attachment to the area, and therefore people feel that they belong to that community (Li & Frieze 2016:776). Belongingness acts as a motivation in human beings because it is one of the basic psychological needs (Timms et al. 2018:244).

Quality means satisfying consumers by fulfilling their felt and perceived needs (Barowski 2013:Online). Quality is an important concept in community engagement, in the sense that it is one of the values promoting community engagement. It is also recognised by the World Grant universities as one of the core values to fuel engagement in higher education (Fitzgerald & Simon 2012:33). However, it can be significantly influenced by the persons leading the community engagement initiatives and their capabilities (Sallnow & Paul 2015:235). Moreover, quality in higher education community engagement is also influenced by the pedagogy undertaken. For example, the work integrated with learning in healthcare settings supports and stimulates the provision of high quality, therefore meeting the current and future care needs of individuals (Pennbrant & Svensson 2018:191). Community engagement needs vigilant planning, communication and organisation (Knight-McKenna et al. 2018:72). This is to ensure that activities are successful and that people participate fully. Schaffer and Hargate (2015:66) noted that work in community settings requires flexibility. This implies that an academic staff member undertaking community engagement activities may be required to change his or her daily schedule several times to carry out activities, as they may not go according to plan because of other commitments of community members. For an academic staff member to exercise flexibility, a range of ways and means are used to engage with the community and to do activities in partnership (Sung & Hepworth 2013:4).

In the health field, there is a saying that ‘if it’s not documented in the medical record then it didn’t happen’. The same is applicable to community engagement. Documentation is necessary for all people involved to keep track of activities. In addition, it will also inform stakeholders what has been accomplished and what needs to be focused on (Crozer Keystone Health 2018:Online). In community engagement such as work-based learning projects, documentation may include the framework, training manual and project report (Fergusson, Allred & Dux 2018:12). For higher education institutions, documentation to explain or summarise how they engaged with the community is necessary (Jacob et al. 2015:154).

### Outcome (consequences) of community engagement

Community engagement leads to more effective health and educational programmes, both in the health sector and at the higher education institution. In the health sector, programmes are effective when they address community challenges, and they help to identify, mobilise and develop relevant community assets and capacity (Sobrero & Jayaratne 2014:140). Effective programmes are needed for an effective health system (Gooden et al. 2013:639). Secondly, the community engagement process develops collaboration. The term ‘engagement’ in ‘community engagement’ refers to collaboration between the university and a targeted community, either at the regional, national or global level (Escrigas et al. 2013:xxxv). This leads to partnership and reciprocity, which is facilitated by a mutually beneficial exchange of knowledge and resources.

Thirdly, community engagement is a process that leads to development in the community and its people. This is because the engaging institutions conduct transactional, transitional and transformational activities that may bring about development in the community and its people. Community development includes a number of areas, covering economic, social, demographic and cultural aspects (Rabinowitz 2018:Online). Fourthly, community engagement leads to integration of teaching, research and services at institutions of higher education. According to the Carnegie Foundation (2015:40), service learning is a method through which community engagement is integrated with teaching and service at the university. This is important because it facilitates the mutual benefit of the students, academic staff and community members. To integrate community engagement with teaching and research, a higher education institution may use a more popular approach called ‘intersecting’. This is practised by incorporating the engagement activities of the department or individual academic staff into either their teaching or research programmes or both (Preece 2017:7). For most higher education institutions, engagement activities are usually in a form of services. Through scholarship of engagement, academic staff and students utilise their knowledge and research to apply to real and pressing social problems and issues, thereby allowing integration of research and teaching to community services (Jacob et al. 2015:116).

**FIGURE 1:**
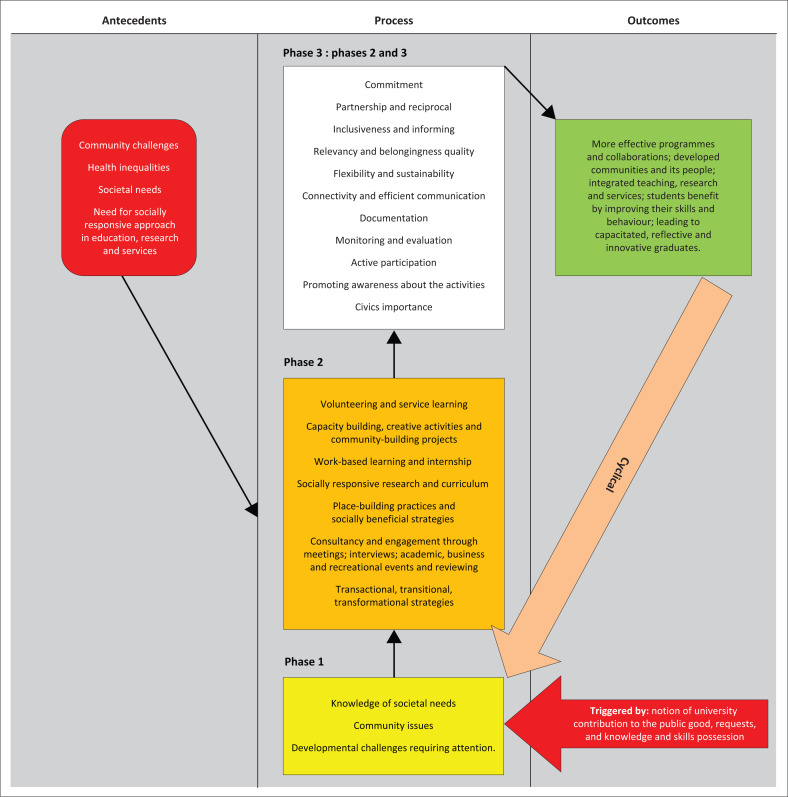
A conceptual map of community engagement.

Fifthly, the community engagement process is an effective indicator of student learning that develops skills such as critical thinking, communication skills, relationships with other students and faculty members as well as overall satisfaction with their learning (Carnegie Foundation 2015:35). Improving skills is facilitated by learning from the community in the process of community engagement. Moreover, service learning, which is one of the forms of community engagement, offers opportunities to cultivate community-centric behaviours in students (Jacob et al. 2015:131). Sixthly, the community engagement process may lead to capacitated, reflective and innovative graduates. For example, in service learning, students are afforded an opportunity to engage with other, different forms of knowledge and experiences beyond what is found in the traditional curriculum (Jacob et al. 2015:168). Capacitated students have the potential to develop into future leaders who are able to tackle health issues in society. It is vital for Health Science programmes to produce reflective graduates because reflection, especially in community engagement, boosts students’ understanding of the programme content, increases obligation to the discipline and promotes a greater sense of civic responsibility (Jacob et al. 2015:159). As a result of exposure to experiential learning pedagogies in community engagement, students tend to be innovative because of the real-life experience. Innovative students are needed in the health field because they are not only prepared for work but may create ideas, leading to a globalised world (Smith-Tolken & Bitzer 2017:30). The above results were used to construct a conceptual map, as displayed in Figure 1. The logic and practical application of community engagement constitutes a cyclical process because it is not a once-off activity but goes through phases. Academic staff members and students need to go through phases, to perform community engagement. Thereafter, they are required to restart the process if one of the antecedents identified in this research is experienced in the community.

## Theoretical definition

Theoretical definitions regularly function as a compressed summary, or outline, of a certain theory (Copi, Cohen & McMahon 2014:88). In this study, the researcher analysed, synthesised and derived the defining attributes of ‘community engagement’ from the findings of a literature review conducted to come up with its theoretical definition:

Community engagement is a core academic function that is undertaken as a multiphased process. It is influenced by community challenges, health inequalities, societal needs and the social responsiveness approach in education and research. It is triggered by the notion of university contributions to the public good, requests, knowledge and skills possession, bringing about scholarly and experiential learning activities for the students, faculty members, community members and partners. It results in more effective programmes and collaborations; *developed communities and peo ple*; integrated teaching, research and services; and improved student skills and behaviour and leads to capacitated, reflective and innovative graduates.

## Implications

The findings of the concept analysis of community engagement have implications for health science education and research. Considering that community engagement is one of the functions of higher education, the theoretical definition developed will help academic staff to gain an understanding of what it constitutes in the Faculty of Health Science. In addition, the theoretical definition developed will be used to develop strategies to facilitate community engagement in the Faculty of Health Science. Lastly, the researcher gained knowledge on concept analysis as a method of inquiry.

## Recommendations

The findings of the concept analysis on community engagement should be used to develop strategies for its facilitation in the Faculty of Health Science at a higher education institution in Namibia.

## Conclusion

Concept analysis as a method of inquiry has the potential to facilitate development of research skills, thus contributing to the professional knowledge and practice advancement (Baldwin & Rose 2009:783). Although concept analysis is regarded as ‘too realistic’ and ‘simplistic’, if sufficiently introduced and correctly used, it has the potential to significantly contribute to theory building (Walker & Avant 2014:179). Despite the availability of guidance on various methods of inquiry, detailed guidelines relating to the different steps of concept analysis are sorely deficient in the existing literature (Schiller 2018:253). This is considered a limitation as researchers interested in undertaking research following concept analysis as a method of inquiry have no detailed step-by-step approach to follow.

This article presents the findings of a concept analysis conducted to clarify the concept *community engagement*. The eight-step model of Walker and Avant was followed in this process to develop the theoretical definition of community engagement. A conceptual map of community engagement was also presented. The researcher has recommended that the conceptual map be utilised in the process of developing strategies for facilitating community engagement.
