# Erratum: Community engagement in the Faculty of Health Science: A concept analysis

**DOI:** 10.4102/hsag.v26i0.1774

**Published:** 2021-12-01

**Authors:** Vistolina Nuuyoma, Agnes Makhene

**Affiliations:** 1 Department of Nursing, Faculty of Health Science, University of Johannesburg, Johannesburg, South Africa

In the version of this article initially published, Nuuyoma, V. & Makhene, A., 2020, ‘Community engagement in the Faculty of Health Science: A concept analysis’, *Health SA Gesondheid* 25(0), 1403. https://doi.org/10.4102/hsag.v25i0.1403, the article section was given incorrectly. The correct section should be Original Research instead of Review Article.

This correction does not alter the study’s findings of significance or overall interpretation of the study’s results. The publisher apologises for any inconvenience caused.

